# A liver secretome gene signature-based approach for determining circulating biomarkers of NAFLD severity

**DOI:** 10.1371/journal.pone.0275901

**Published:** 2022-10-19

**Authors:** Christoffer A. Hagemann, Christian Legart, Mathias B. Møllerhøj, Martin R. Madsen, Henrik H. Hansen, Merete J. Kønig, Frederik Helgstrand, Flemming P. Hjørne, Anders Toxværd, Jill L. Langhoff, Urd L. Kielgast, Lise L. Gluud, Helene Ægidius, Kristoffer T. G. Rigbolt, Tina Vilsbøll, Jacob Jelsing, Filip K. Knop

**Affiliations:** 1 Gubra, Hørsholm, Denmark; 2 Center for Clinical Metabolic Research, Copenhagen University Hospital—Herlev and Gentofte, Copenhagen, Denmark; 3 Department of Radiology, Copenhagen University Hospital—Herlev and Gentofte, Copenhagen, Denmark; 4 Department of Gastrointestinal Surgery, Zealand University Hospital, Køge, Denmark; 5 Department of Pathology, Copenhagen University Hospital—Herlev and Gentofte, Copenhagen, Denmark; 6 Department of Medicine, Zealand University Hospital, Køge, Denmark; 7 Gastro Unit, Medical Division, Copenhagen University Hospital—Amager and Hvidovre, Copenhagen, Denmark; 8 Department of Clinical Medicine, University of Copenhagen, Copenhagen, Denmark; 9 Steno Diabetes Center Copenhagen, Gentofte, Denmark; 10 Novo Nordisk Foundation Center for Basic Metabolic Research, University of Copenhagen, Copenhagen, Denmark; Medizinische Fakultat der RWTH Aachen, GERMANY

## Abstract

Non-invasive biomarkers of non-alcoholic fatty liver disease (NAFLD) supporting diagnosis and monitoring disease progression are urgently needed. The present study aimed to establish a bioinformatics pipeline capable of defining and validating NAFLD biomarker candidates based on paired hepatic global gene expression and plasma bioanalysis from individuals representing different stages of histologically confirmed NAFLD (no/mild, moderate, more advanced NAFLD). Liver secretome gene signatures were generated in a patient cohort of 26 severely obese individuals with the majority having no or mild fibrosis. To this end, global gene expression changes were compared between individuals with no/mild NAFLD and moderate/advanced NAFLD with subsequent filtering for candidate gene products with liver-selective expression and secretion. Four candidate genes, including *LPA* (lipoprotein A), *IGFBP-1* (insulin-like growth factor-binding protein 1), *SERPINF2* (serpin family F member 2) and *MAT1A* (methionine adenosyltransferase 1A), were differentially expressed in moderate/advanced NAFLD, which was confirmed in three independent RNA sequencing datasets from large, publicly available NAFLD studies. The corresponding gene products were quantified in plasma samples but could not discriminate among different grades of NAFLD based on NAFLD activity score. *Conclusion*: We demonstrate a novel approach based on the liver transcriptome allowing for identification of secreted hepatic gene products as potential circulating diagnostic biomarkers of NAFLD. Using this approach in larger NAFLD patient cohorts may yield potential circulating biomarkers for NAFLD severity.

## Introduction

Non-alcoholic fatty liver disease (NAFLD) is an increasing health care problem driven by the obesity and diabetes pandemic, currently affecting 20–25% of the adult population [1]. NAFLD encompasses a broad spectrum of liver diseases ranging from simple steatosis over non-alcoholic steatohepatitis (NASH) with or without fibrosis to end-stage cirrhosis with liver failure [2]. Whereas hepatic steatosis alone (non-alcoholic fatty liver (NAFL)) is considered a low-risk reversible condition, NASH is a progressive condition causing hepatic fibrosis increasing the risk of overt cirrhosis [1, 3]. The pathogenesis of NAFLD is closely associated with components of the metabolic syndrome, notably obesity, type 2 diabetes, and hypertension [1, 2].

Although several non-invasive surrogate markers for ruling out advanced liver fibrosis have been developed [4], liver biopsy histology remains the gold standard for confirming the diagnosis of NASH and the extent of fibrosis [2, 5]. Assessment of NAFLD activity score (NAS), a composite score of steatosis, lobular inflammation and ballooning degeneration, is often used for semiquantitative assessment of disease activity, allowing comparison of liver biopsies in clinical studies [6]. Given the often asymptomatic cause of the disease and the limitations associated with the invasive and costly biopsy procedure, many NAFLD patients are not diagnosed until progression to advanced disease with severe liver complications. This has spurred a search for non-invasive biomarkers to support diagnosis and treatment intervention at earlier disease stages.

Despite increasing understanding of the molecular changes underlying NAFLD progression, reliable noninvasive biomarkers for diagnosing NAFLD remain to be identified [7, 8]. Mass spectrometry-based approaches have previously predicted biomarkers from the plasma proteome of patients with varying degrees of NAFLD [9]. These approaches are, however, hampered by the complexity of the blood proteome, and a limited detection rate and dynamic range of the mass spectrometry technologies applied [10, 11]. Human liver transcriptome studies over the past two decades have provided important insight into the complex molecular pathways involved in the pathogenesis of NAFLD/NASH [12–15]. Liver biopsy gene expression patterns in NAFLD may therefore provide a basis for defining proteins/peptides secreted into the circulation that may serve as more reliable biomarkers of NAFLD/NASH [14].

Here, we set out to profile liver secretome gene expression changes in severely obese patients with NAFLD with the aim to predict and identify potential novel circulating diagnostic biomarkers of NAFLD progression.

## Materials and methods

### Regulatory approvals and registrations

The study was approved by the Scientific-Ethical Committee of the Capital Region of Denmark (registration number H-18059758) and the Danish Data Protection Agency (approval number P-2019-56). The study is registered at ClinicalTrials.gov (identifier NCT04043585) and was conducted in accordance with the Declaration of Helsinki (7th revision, 2013). Written informed consent was obtained from all participants before inclusion.

### Study participants

Twenty-six individuals referred to Roux-en-Y gastric bypass or sleeve gastrectomy at the Department of Gastrointestinal Surgery, Zealand University Hospital (Køge, Denmark), were included in the study. Study participants were also part of an ongoing prospective study investigating the effects of bariatric surgery on NAFLD (ClinicalTrials.gov identifier: NCT04043585). Study eligibility criteria included general criteria for bariatric surgery according to the Danish national bariatric guidelines; body mass index (BMI) greater than 40 kg/m^2^ or a BMI greater than 35 kg/m^2^ together with one or more obesity-related comorbidities (type 2 diabetes, uncontrolled hypertension, lower-extremity arthrosis, sleep apnea or infertility and pregnancy desire in women with polycystic ovarian syndrome). In the present study, study inclusion was further based on the evidence of potential NASH with fibrosis (fibrosis-4 score ≥ 1.30, NAFLD fibrosis score ≥ −1.455, or transient elastography ≥ 7.0 kPa [16–18]). Participants with an excess alcohol intake over a two-year period prior to inclusion (≥20 g/day for women and ≥ 30 g/day for men [2, 4]), use of medication known to induce NAFLD, or pre-existing liver disease other than NAFLD were excluded from participation. All participants were screened for other etiologies of liver disease including hepatitis B and C, alpha-1 antitrypsin deficiency and hemochromatosis. At the time of inclusion, 10 participants were diagnosed with type 2 diabetes, 12 participants were diagnosed with hypertension and 12 participants were diagnosed with

dyslipidemia (Table 1).

**Table 1:** Clinical, anthropometrical and biochemical characteristics of study participants divided into three groups with increasing NAS.

	NAS 0–1 (*n* = 8)	NAS 2–3 (*n* = 12)	NAS 4–6 (*n* = 6)	*p-v*alue
Age (years)	54 (44;57)	48 (32;53)	48 (40;55)	0.333
Sex (male/female)	6/2	3/9	4/2	-
Type 2 diabetes (*n*)	4	3	3	-
Hypertension (*n*)	3	5	4	-
Dyslipidemia (*n*)	5	2	5	-
Weight (kg)	137 (112;151)	149 (138;170)	153 (138;159)	0.273
BMI (kg/m^2^)	41.9 (39.1;48.3)	51.8 (47.5;55.9)*	47.3 (42.0;51.3)	0.023
Lean body mass, DXA (kg)	71.4 (50.6;76.4)	60.9 (57.2;75.8)	77.0 (66.1;83.5)	0.340
Fat mass, DXA (kg)	62.5 (54.0;66.2)	81.0 (67.5;90.7)*	66.8 (60.5;72.9)	0.030
Body fat, DXA (%)	46.5 (44.2;49.9)	54.5 (51.5;56.1)*	46.7 (42.8;48.8)	0.002
Waist/hip ratio	1.01 (0.86;1.14)	0.91 (0.87;1.03)	1.03 (1.01;1.11)	0.129
Transient elastography (kPa)	4.6 (3.4;8.7)	9.0 (6.1;10.5)	13.4 (9.3;17.2)**	0.016
Transient elastography (CAP)	363 (269;371)	348 (299;385)	376 (354;389)	0.214
Fibrosis-4 score (points)	0.61 (0.48;0.74)	0.60 (0.46;0.69)	0.84 (0.50;1.38)	0.442
NAFLD fibrosis score (points)	-0.98 (-1.07;-0.37)	-1.03 (-1.70;-0.22)	-0.99 (-1.56;0.80)	0.918
ALT (U/L)	27 (17;34)	41 (32;48)	60 (53;72)**	0.005
AST (U/L)	16 (14;22)	26 (22;28)	40 (25;53)**	0.008
GGT (U/L)	30 (19;40)	39 (31;45)	56 (33;103)	0.165
Glucose (mmol/L)	6.1 (5.3;8.4)	6.0 (5.4;6.4)	6.4 (6.1;8.3)	0.215
HbA_1c_ (mmol/mol)	41 (36;51)	37 (34;40)	45 (38;62)	0.070
Insulin (pmol/L)	163 (57;1390)	150 (116;248)	218 (162;494)	0.408
C-peptide (pmol/L)	553 (206;1,010)	962 (854;1,240)	1,290 (988;1,705)**	0.010
Insulin resistance, HOMA-2	1.2 (0.6;2.4)	2.3 (2.0;3.0)	3.1 (2.5;4.1)**	0.005
LDL (mmol/L)	2.8 (2.2;3.8)	2.4 (1.9;2.8)	3.2 (2.5;3.6)	0.267
HDL (mmol/L)	1.0 (0.9;1.4)	1.0 (0.8;1.4)	1.1 (1.0;1.2)	0.804
VLDL (mmol/L)	0.9 (0.5;1.0)	0.7 (0.6;0.7)	0.5 (0.5;0.7)	0.191
Triglycerides (mmol/L)	1.9 (1.2;2.3)	1.4 (1.3;1.6)	1.2 (1.1;1.6)	0.261
hsCRP (mg/L)	5.7 (2.8;11.1)	9.2 (4.7;11.7)	5.7 (2.6;8.4)	0.218

Data are presented as median with interquartile range in parentheses unless otherwise stated. P-values are Kruskal-Wallis tests.

* *p* <0.05

** *p* <0.01 (Dunn’s post hoc test compared to NAS 0–1) Abbreviations: NAFLD; non-alcoholic fatty liver disease; NAS, NAFLD activity score; BMI, body mass index; CAP, controlled attenuation parameter; ALT, alanine aminotransferase; AST, aspartate aminotransferase; GGT, gamma-glutamyl transferase; HbA1c, glycated hemoglobin A1c; HOMA, homeostasis model assessment; LDL, low-density lipoprotein; HDL, high-density lipoprotein; VLDL, very-low density lipoprotein; hsCRP, high-sensitive C-reactive protein.

### Study design

Participants attended two study days with a maximum of four days between visits. All study days were scheduled before the start of the dietician-monitored, diet-induced weight loss of minimum 8% required before government-sponsored bariatric surgery in Denmark. The study was conducted from July 2019 to January 2021 at Center for Clinical Metabolic Research, Copenhagen University Hospital–Herlev and Gentofte (Copenhagen, Denmark). Both study days were preceded by a minimum of 10 hour fast including liquids and medications and refrainment from tobacco use. On day one, anthropometrics and patient characteristics were registered. Fasting blood samples, a urine sample for assessment of albuminuria and human chorionic gonadotropin when relevant, liver stiffness measured by transient elastography (FibroScan^®^, XL probe), bioimpedance analysis and a questionnaire to evaluate alcohol consumption were collected [19]. On day two, a percutaneous liver biopsy was sampled as described below. A full body dual-energy X-ray absorptiometry (DXA) scan was performed on either day one or two. The same investigator was responsible for all experimental days.

### Liver biopsy and histological examination

Two ultrasound-guided percutaneous liver biopsies were sampled under local anesthesia from the right liver lobe using an 18-gauge needle and performed according to national and international guidelines by the same trained radiologist [20]. A liver biopsy specimen of 17 mm in length was sent for histopathological assessment and two specimens of ~9 mm in length were snap-frozen in liquid nitrogen at bedside immediately after collecting the liver biopsy. Snap-frozen liver tissue was subsequently stored at −80°C until RNA sequencing. The liver tissue for histopathological assessment was fixed in neutrally buffered formalin at room temperature for 24–48 hours, embedded in paraffin, and cut in slides according to standard pathology guidelines. Standardized histopathologic assessment was performed independently by the same two experienced pathologists at Department of Pathology, Copenhagen University Hospital–Herlev and Gentofte (Copenhagen, Denmark) blinded to patient data. NAFLD was assessed by the NAS as the sum of scores for steatosis, lobular inflammation and hepatocellular ballooning. In the event of score disagreement, a consensus between the two pathologists was sought. Liver fibrosis was staged from F0 to F4 (stages F1a, F1b, and F1c were considered as F1) using the CRN criteria outlined by Kleiner et al. [6]. The histological criteria for NASH were defined by the presence of steatosis together with hepatocellular ballooning and lobular inflammation with or without fibrosis. Participants were subsequently divided into three groups based on composite NAS: NAS 0–1 (no or mild NAFLD), 2–3 (moderate NAFLD) and 4–6 (more advanced NAFLD). Given the study cohort size, the NAS intervals applied allowed for assessment of gene expression changes with reference to various degrees of NAFLD.

### DXA scan

A single DXA scanner was used for all body composition scans (Lunar iDXA, GE Healthcare, Madison, Wisconsin, USA) and data were analyzed using the Encore software version 17 (GE Healthcare, Chicago, Illinois, USA). Lean body mass was estimated by subtracting fat and bone mass from total body weight. Fat mass in percent was calculated as the total amount of fat divided by total body weight. Two participants did not undergo DXA scans.

### RNA sequencing

Analysis of the liver transcriptome was performed by RNA sequencing of RNA extracts from human liver biopsies (*n* = 26). RNA was extracted and purified with NucleoSpin^®^ 8 RNA Core Kit (Macherey-Nagel, Düren, Germany). The RNA quantity was measured using Qubit^®^ (Thermo Scientific, Eugene, Oregon, USA). A total of 100 ng purified RNA from each sample was used to generate a cDNA library using the NEBNext^®^ Ultra^™^ II Directional RNA Library Prep Kit for Illumina (New England Biolabs, Ipswich, Massachusetts, USA). cDNA libraries were sequenced 75 cycles on a NextSeq 500 using NextSeq 500/550 High Output Kit V2 (Illumina, San Diego, California, USA) to a depth of ~15 million reads per library. Reads were mapped to the Ensembl human genome (GRCh38.p10) using STAR v.2.7.0f with default parameters. The R package DESeq2 v.1.24.0 [21] was used for differential expression analysis. *p* values were adjusted by the Benjamini-Hochberg method with 0.05 as the cut-off. In the comparison between patient groups, the Reactome pathway database [22] was used as gene annotation using the R package PIANO v.1.18.1 [23], with the Stouffer method and Benjamini-Hochberg adjusted *p* values.

### Candidate gene selection procedure

**Fig 1:**
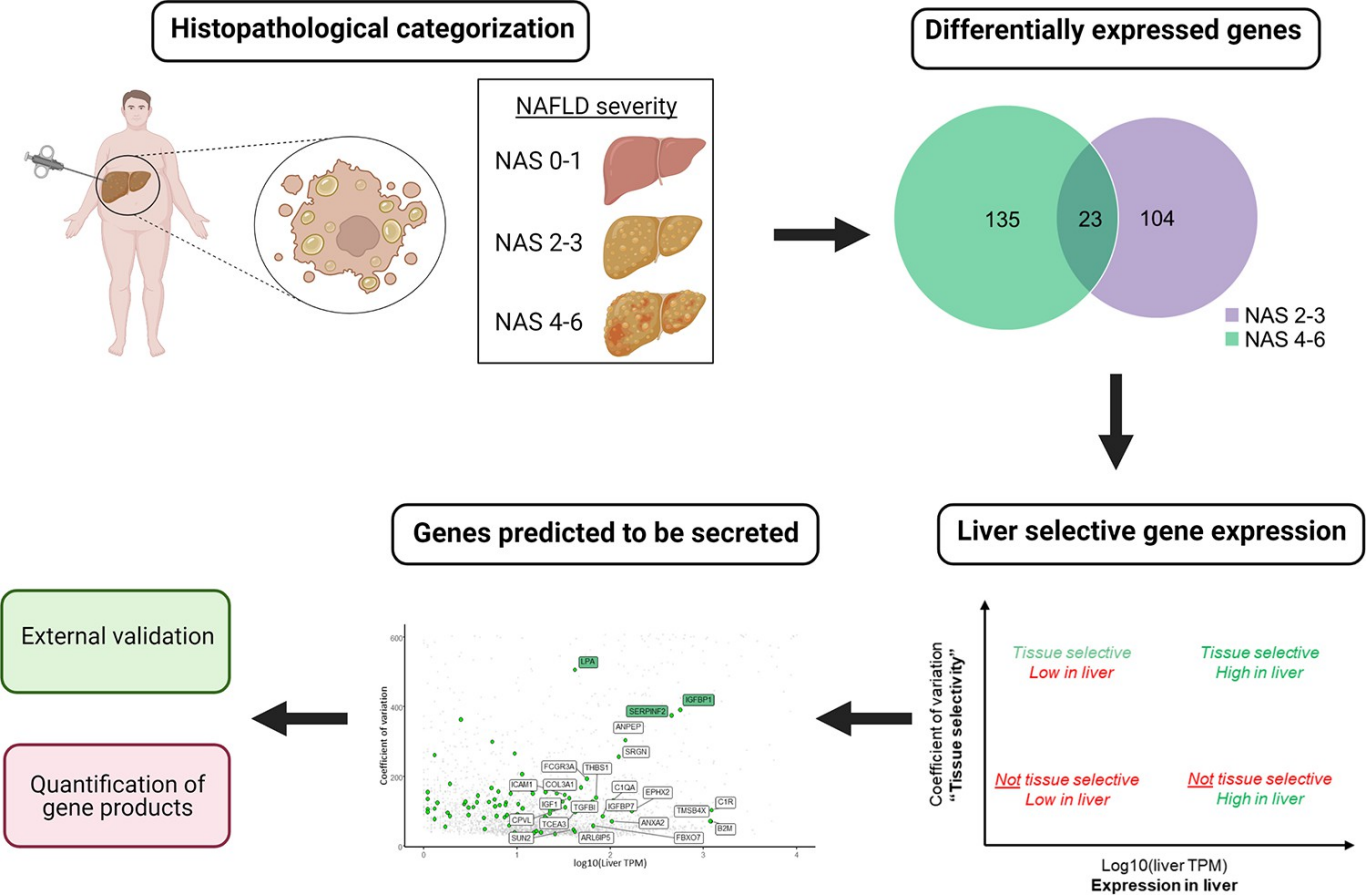
Overview of the concept for identifying potential, circulating biomarkers of NAFLD. Liver secretome gene signatures were generated from DEGs between individuals with no/mild NAFLD and moderate to advanced NAFLD with subsequent filtering for candidate gene products with liver-selective expression and secretion. Abbreviations: DEGs, differentially expressed genes; NAFLD, non-alcoholic fatty liver disease; NAS, NAFLD activity score. Created with BioRender.com.

We assumed three general gene characteristics of an applicable biomarker of NAFLD: 1) the gene product is selectively expressed in the liver; 2) the gene is differentially expressed in individuals with moderate to more advanced NAFLD compared to no/mild NAFLD; and 3) the gene product is secreted from liver cells into circulation. Biomarker prediction relied on a stepwise bioinformatics approach (Fig 1).

First, differentially expressed genes (DEGs) between individuals with no/mild NAFLD and individuals with moderate and more advanced NAFLD were identified as outlined above. Secondly, we evaluated the degree to which genes were liver-selectively expressed. For this, we referenced gene expression data from the human protein atlas [24] and evaluated liver expression levels relative to the coefficient of variation across all tissues (see the Results section for further details). A high coefficient of variation indicates large differences in expression across tissues and implies that a gene is selectively expressed in a few or a single tissue. Liver selective genes would thus display high liver gene expression in combination with high coefficient of variation. A candidate gene was considered if the corresponding gene product had been reported secreted from cultured human hepatocytes [25], detected in any human blood proteome dataset [24] or predicted as secreted according to a curated list of secreted human proteins [26] (Fig 1).

### Plasma analyses

Fasted blood was collected in K_2_EDTA or K_3_EDTA tubes. All tubes were immediately cooled on ice, centrifuged for 15 minutes at 2,000 *g* and 4°C and stored at −80°C until analysis. ELISA kits were used for assessment of human lipoprotein A (LPA, #ab212165, Abcam, Cambridge, UK), insulin-like growth factor-binding protein 1 (IGFBP-1, #ab213789, Abcam, Cambridge, UK), alpha-2 antiplasmin (α2AP, #ab254502, Abcam, Cambridge, UK) and S-adenosylmethionine (SAM, #MET-5152, Cell Biolabs Inc., San Diego, California, USA). All biomarker assays were measured according to the manufacturer’s instructions.

### Quantitative histology

Remaining paraffin-embedded liver tissue was subsequently cut into 3 μm sections at Gubra (Hørsholm, Denmark). Sections were stained with hematoxylin-eosin (HE), picro-Sirius Red (PSR, Sigma-Aldrich, Brøndby, Denmark), anti-galectin-3 (cat. 125402, Biolegend, San Diego, California, USA), alpha-smooth muscle action (α-SMA, cat. ab124964, Abcam, Cambridge, UK), anti-type I collagen (COL1A1, cat. 1310–01, Southern Biotech, Birmingham, Alabama, USA), CD11b (cat. ab133357, Abcam, Cambridge, UK) or CD45 (cat. ab10558, Abcam, Cambridge, UK) using standard procedures. A digital imaging software (Visiomorph^®^, Visiopharm, Hørsholm, Denmark) was used for quantification of whole-section liver fat (HE-staining), fibrosis (PSR, COL1A1), inflammation (galectin-3, CD11b, CD45) and hepatic stellate cell activation (α-SMA). Histochemical positive staining areas were expressed relative to total tissue sectional area (fractional area, in percent).

### Statistical analysis

Results are presented as mean ± standard deviation unless otherwise stated. Insulin resistance was evaluated by homeostasis model assessment (HOMA) based on fasting plasma concentrations of glucose and C-peptide (HOMA2 calculator https://www.dtu.ox.ac.uk/homacalculator/). Since the criteria for normal distribution were not met, non-parametric testing was carried out. Comparison between the three patient groups assessing clinical, anthropometrical, biochemical and quantitative histological data were analyzed using Kruskal-Wallis one-way analysis of variance (ANOVA) test. Dunn’s post hoc test was used for comparison of data from patient groups NAS 2–3 and NAS 4–6 with NAS 0–1. Correlations were analyzed by Spearman’s correlation. Statistical analyses were carried out using GraphPad Prism v9.1.1 (GraphPad Software, San Diego, California, USA). A *p* value less than 0.05 was considered statistically significant.

## Results

### More advanced NAFLD correlates with greater insulin resistance and clinical signs of liver disease

Clinical, anthropometrical and biochemical features of the study population are summarized in Table 1 and S1 Table, and histopathological features are summarized in Table 2.

**Table 2:** Frequency table of NAS, fibrosis grade and NASH of study participants divided into three groups with increasing NAS.

	NAS 0–1 (*n* = 8)	NAS 2–3 (*n* = 12)	NAS 4–6 (*n* = 6)
Steatosis grade (0/1/2/3)	3/5/0/0	0/6/5/1	0/0/3/3
Inflammation grade (0/1/2/3)	8/0/0/0	4/8/0/0	0/6/0/0
Ballooning grade (0/1/2)	8/0/0	9/3/0	0/5/1
NAS total sum (0/1/2/3/4/5/6/7/8)	3/5/0/0/0/0/0/0/0	0/0/6/6/0/0/0/0/0	0/0/0/0/3/2/1/0/0
Fibrosis grade (0/1/2/3/4)	8/0/0/0/0	9/3/0/0/0	1/2/2/1/0
NASH (no/yes)	8/0	12/0	1/5

Data are presented as frequencies of each NAS sub-score. Abbreviations: NAS, non-alcoholic fatty liver disease activity score; NASH, non-alcoholic steatohepatitis.

Steatosis was the strongest determinant for patient grouping according to NAFLD activity scores (NAS 0–1, NAS 2–3, NAS 4–6). Only 5 out of 26 individuals were diagnosed with NASH. Liver fibrosis (stage 1–2) was observed in 8 individuals, all having NAS ≥2 (NAS 2–3, n = 3; NAS 4–6, n = 5) (Table 2). Individuals with more advanced NAFLD (NAS 4–6) were more insulin-resistant, reflected by increased HOMA and C-peptide levels, and had higher levels of plasma markers of liver injury (alanine aminotransferase, aspartate aminotransferase, lactate dehydrogenase) compared to individuals with no/mild NAFLD. Moreover, liver stiffness assessed by transient elastography was increased in individuals with more advanced NAFLD compared to individuals with no/mild NAFLD. In contrast, there was no difference in the Fibrosis-4 score or the NAFLD fibrosis score between the three NAFLD categories. Individuals with moderate NAFLD (NAS 2–3) had the highest BMI and adiposity as assessed by DXA scan and bioimpedance (Table 1 and S1 Table). Thus, in agreement with previous findings [27], total fat mass *per se* did not appear to be a determinant of advanced NAFLD.

### Comparison of NAFLD activity scores and quantitative histological hallmarks

Individuals with NAS 2–3 and NAS 4–6 demonstrated increased proportional (%) area of fat (S1 Fig), consistent with NAFLD severity predominantly being driven by steatosis. A higher NAFLD score was also paralleled by increased proportional area of PSR staining for fibrosis (S1 Fig). Only individuals with NAS 4–6 showed significantly elevated PSR levels, corresponding to a higher rate of fibrosis in this group. In contrast, immunohistochemical markers of inflammation (CD11b), macrophage infiltration (CD45, Galectin-3) and fibrogenesis (COL1A1, αSMA) were not significantly elevated in individuals with moderate or more advanced NAFLD as compared to individuals with no/mild NAFLD (S2 and S3 Figs). There was no correlation between quantitative levels of immunohistochemical markers and corresponding gene expression levels of *LGALS3*, *ITGAM*, *PTPRC*, *a-SMA*, *COL1A1* (S2 Fig).

### Global gene expression patterns clearly separate individuals with no or mild NAFLD from individuals with moderate and more advanced NAFLD

**Fig 2:**
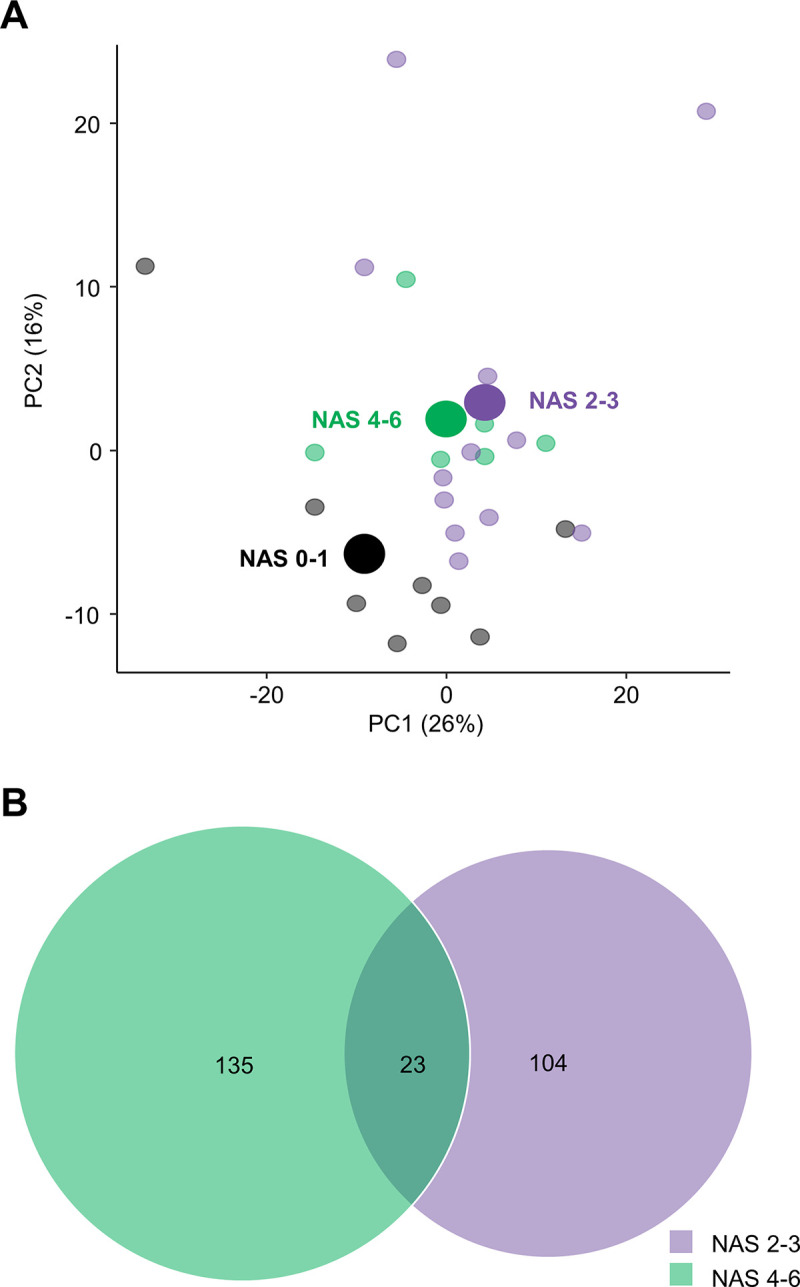
Patients with moderate and more advanced NAFLD cluster together separating from patients with no or mild NAFLD. **(A)** PCA plot of samples based on 500 most variable genes. Points indicate the relationship between liver biopsy samples (patients) across their gene expression profile. Color represent group defined by NAS score. **(B)** Venn diagram of DEGs compared to individuals with NAS 0–1 (false discovery rate, *p* <0.05). Circles that overlap share DEGs. Abbreviations: DEGs, differentially expressed genes; NAFLD; non-alcoholic fatty liver disease; NAS, NAFLD activity score; PCA, principal component analysis.

A principal component analysis (PCA) was applied to obtain an overview of liver biopsy transcriptome signatures in the three groups based on composite NAS. The PCA plot illustrates that global gene expression profiles in individuals with moderate (NAS 2–3) and more advanced (NAS 4–6) NAFLD clustered together and were clearly separated from individuals with no/mild NAFLD (NAS 0–1) (Fig 2A).

**Fig 3:**
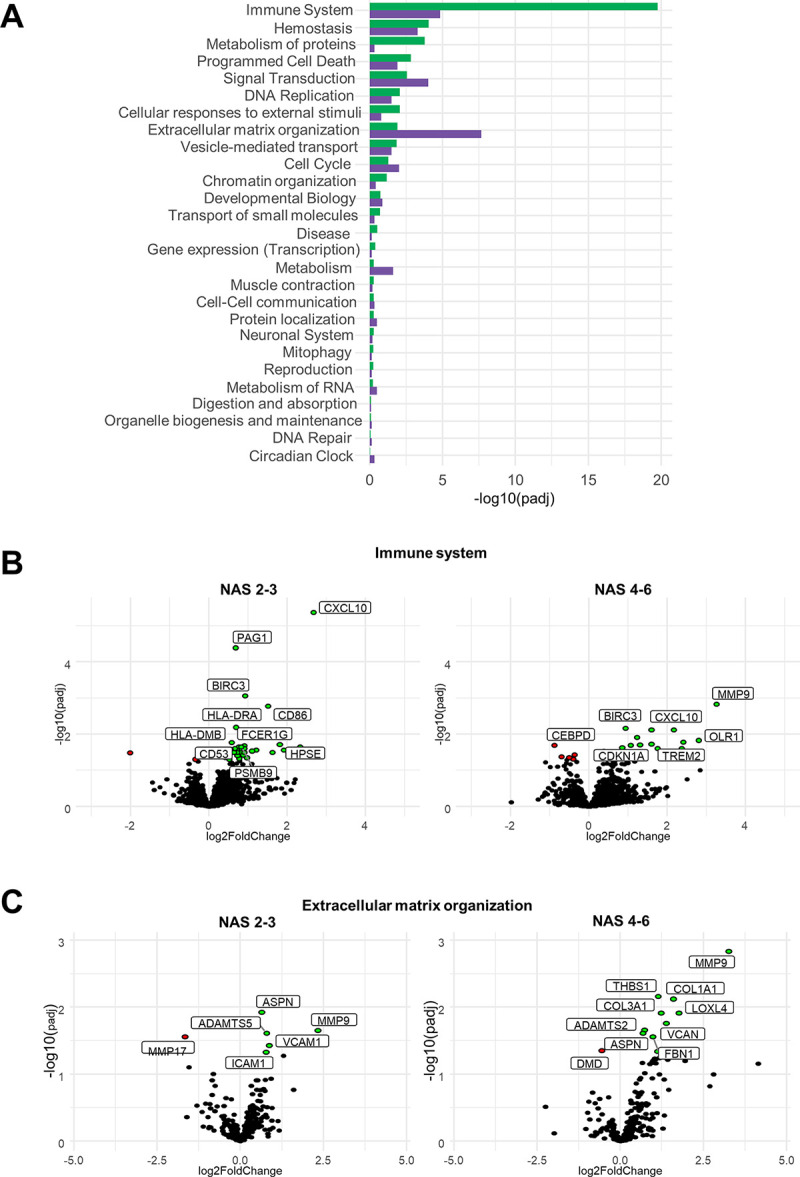
Progression of NAFLD is accompanied by a change in immune system and extracellular matrix organization pathways. **(A)** Overview of differentially regulated Reactome pathways (log10 of false discover rate) in individuals with NAS 2–3 and NAS 4–6 as compared to individuals with no/mild NAFLD (NAS 0–1). **(B, C)** Volcano plots illustrating DEGs in selected Reactome pathways, i.e., immune system and extracellular matrix organization Reactome pathways compared to individuals with no/mild NAFLD (NAS 0–1). Significantly regulated genes (*p* <0.05) are indicated in red (downregulation) and green (upregulation), respectively. Abbreviations: DEGs, differentially expressed genes; NAFLD; non-alcoholic fatty liver disease; NAS, NAFLD activity score.

Compared to no/mild NAFLD, individuals with moderate and more advanced NAFLD demonstrated 127 and 158 DEGs, respectively (Fig 2B). Comparing moderate to more advanced NAFLD, only 15 DEGs (*VCAN*, *IGFALS*, *MDGA1*, *DPPA4*, *COL10A1*, *PPP1R1A*, *PLK2*, *MAT1A*, *NDST3*, *PACSIN3*, *HACD1*, *TMC4*, *ZMAT3*, *OR7D2*, *FAM166A*) were differentially expressed indicating high similarity in global gene expression. A Reactome pathway enrichment analysis revealed that several DEGs in individuals with moderate and more advanced NAFLD, as compared to individuals with no/mild NAFLD, were associated with the immune system (upregulated: *BIRC3*, *CD53*, *CD86*, *CXCL10*, *FCER1G*, *HLA-DRA*, *HLA-DMB*, *HPSE*, *PAG1*, *PSMB9*) and extracellular matrix organization (upregulated: *ADAMTS5*, *ASPN*, *COL1A1*, *COL3A1*, *ICAM1*, *LOXL4*, *MMP9*, *THBS1*, *VCAN*, *VCAM1; downregulated DMD*, *MMP17*,,) (Fig 3).

### Liver secretome gene signatures predict several secreted gene products as potential biomarkers of NAFLD

**Fig 4:**
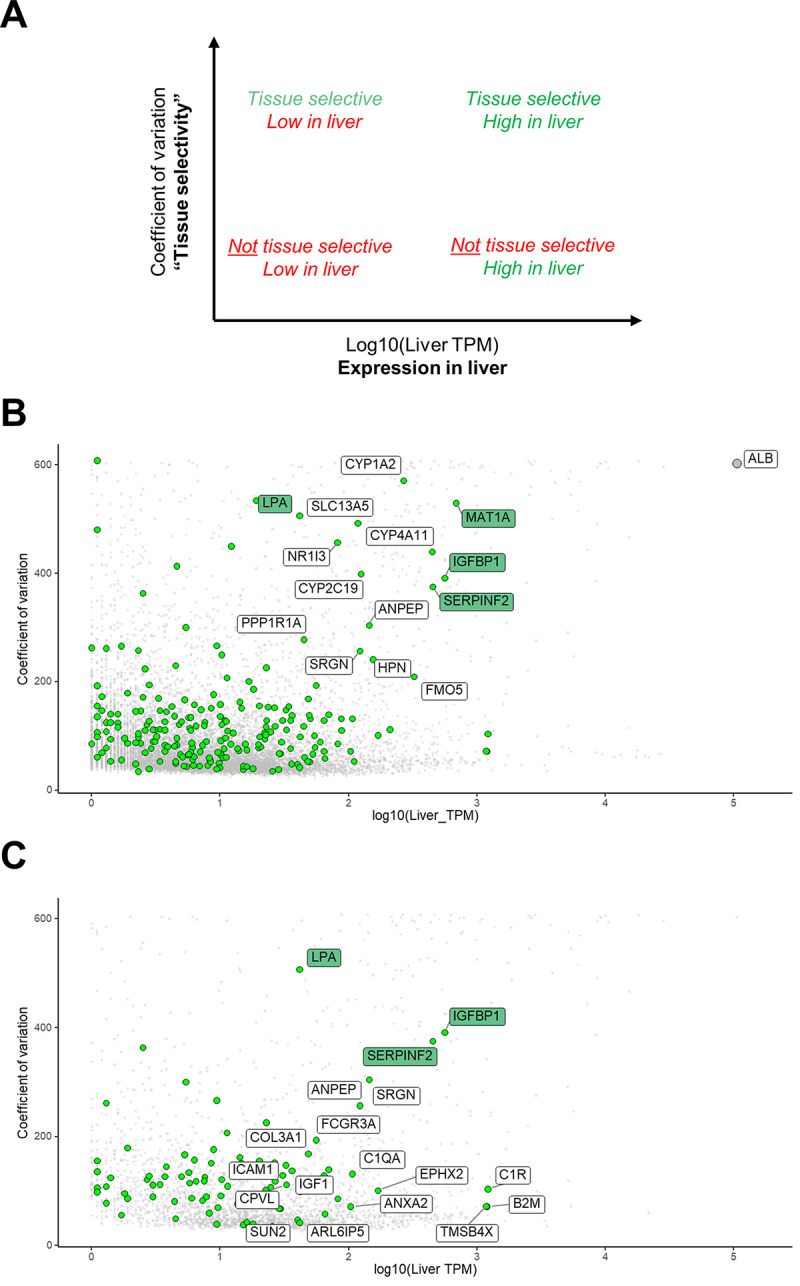
Selection of candidate genes with biomarker potential. **(A)** Evaluation of gene expression by coefficient of variation across all tissues (y-axis) versus liver tissue expression levels (x-axis, TPM). A high coefficient of variation with respect to tissue selectivity in combination with high liver expression (towards the top right corner) suggests liver-selective expression. **(B)** Coefficient of variation versus liver tissue expression levels for all identified genes (grey dots) including all DEGs (green dots) as well as for albumin. **(C)** Coefficient of variation versus liver tissue expression levels for all identified genes (grey dots) including all DEGs (green dots) that were predicted as secreted. Highlighted genes (green boxes), LPA (lipoprotein A), IGFBP-1 (insulin-like growth factor-binding protein 1), SERPINF2 (serpin family F member 2) and MAT1A (methionine adenosyltransferase 1A), were selected and evaluated as gene biomarker candidates of NAFLD. Abbreviations: ALB, albumin; DEGs, differentially expressed genes; NAFLD; non-alcoholic fatty liver disease; TPM, transcript per million.

Gene expression data from the human protein atlas [24] were evaluated for hepatic global gene expression levels relative to the coefficient of variation across all tissues. A high coefficient of variation in combination with high liver expression suggests that expression of the relevant gene is highly liver-selective, as illustrated in Fig 4A.

**Fig 5:**
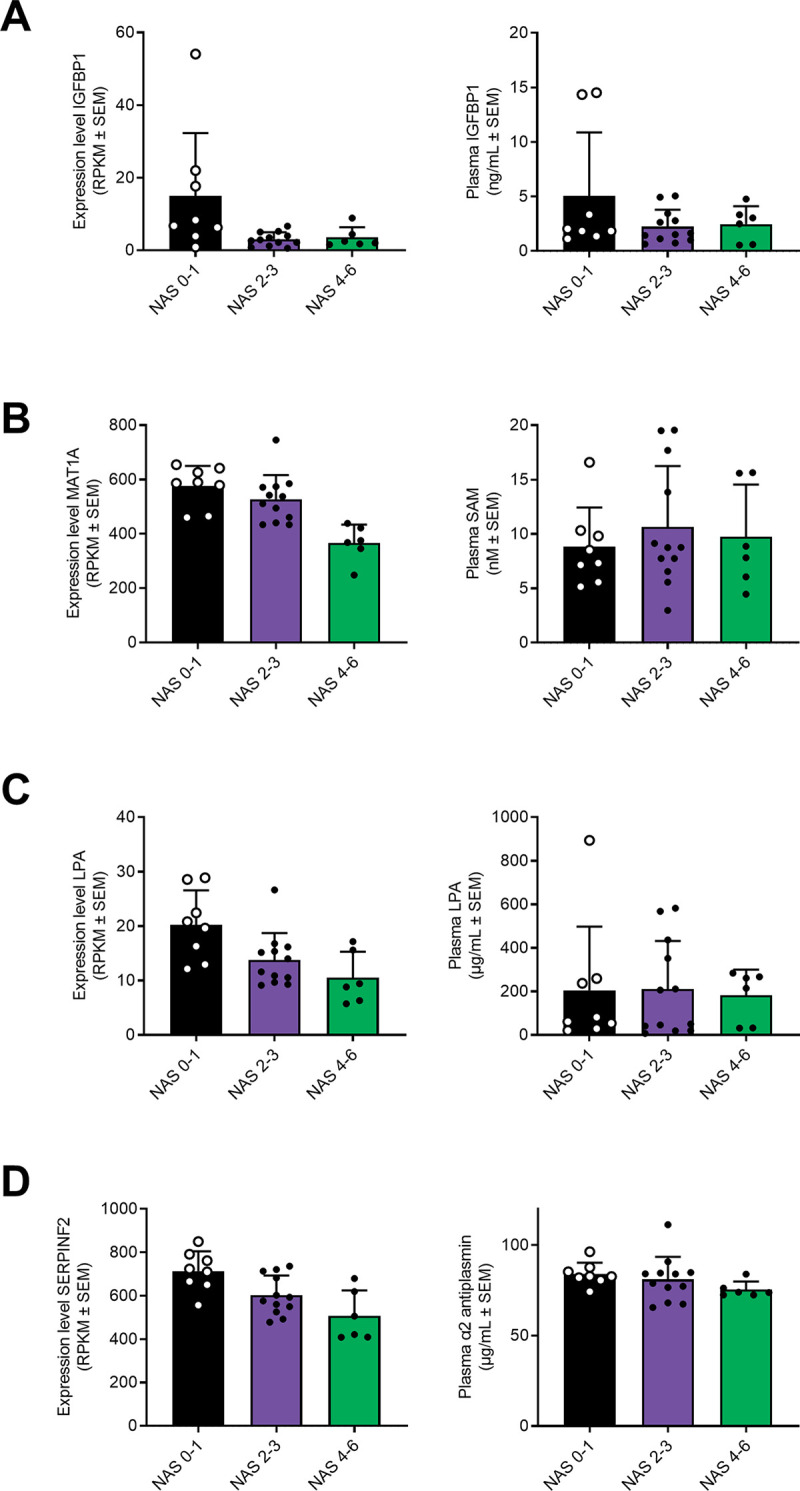
Hepatic candidate gene expression levels compared to plasma concentrations of the corresponding gene product. Left panels, gene expression levels. Right panels, plasma concentrations of the corresponding gene product. **(A)** Insulin-like growth factor-binding protein 1 (IGFBP-1). **(B)** Methionine adenosyltransferase 1A (MAT1A) and enzyme catalytic product (S-adenosylmethionine (SAM)). **(C)** Lipoprotein A (LPA). **(D)** Serpin family F member 2 (SERPINF2) and gene product alpha-2 antiplasmin (α2AP). * *p* <0.05, ** *p* <0.01 vs. NAS 0–1.

The coefficient of variation versus liver tissue expression levels was mapped for all hepatic genes, including those differentially expressed between patient groups (Fig 4B). DEGs positioned towards the top right corner suggested differential expression of liver-selective genes. DEGs with potentially liver-secreted gene products (*n* = 97) are depicted in Fig 4C. Based on this stepwise bioinformatics approach, we predicted *LPA* (lipoprotein A), *IGFBP-1* (insulin-like growth factor-binding protein 1) and *SERPINF2* (serpin family F member 2) as potentially secreted biomarkers with the highest liver-selectivity (Fig 4C). Also, *MAT1A* (methionine adenosyltransferase 1A) displayed a liver-selective expression profile, although, not predicted to be secreted (Fig 4B). However, *MAT1A* encodes a catalytic subunit involved in the first step in the methionine metabolism in which methionine and adenosine triphosphate are converted to S-adenosylmethionine (SAM) which previously has been detected in the circulation [28]. We, therefore, hypothesized that differences in *MAT1A1* expression could translate into differences in plasma SAM levels, thereby serving as an indirect marker of MAT1A activity. Hepatic gene expression levels of *LPA*, *IGFBP-1*, *SERPINF2* and *MAT1A* are depicted in Fig 5 (left panel).

Hepatic DEGs and secretome gene profiles between different NAFLD categories based on NAS were confirmed in RNA sequencing datasets from three large, publicly available NAFLD studies [13–15] (S4 Fig). Candidate genes displayed high levels of expression in the liver and low expression in few or no other tissues (S5 Fig).

### Selected candidate gene products do not discriminate among different NAFLD categories

Commercially available ELISA kits were used to quantify the respective candidate gene products LPA, IGFBP-1, α2AP (*SERPINF2*) and SAM (*MAT1A*) in the plasma samples. Overall, gene product levels in plasma were unable to discriminate among different NAFLD categories based on NAS (Fig 5, right panel). In agreement, no correlation was observed for *LPA*, *IGFBP-1*, *SERPINF2* and *MAT1A* vs. their corresponding plasma proteins (S6 Fig). Furthermore, there was no correlation between plasma concentrations of the individual candidate gene products and insulin resistance as assessed by HOMA. However, plasma IGFBP-1 correlated with age (r = 0.47, *p* = 0.015) and plasma C-peptide concentrations (r = −0.48, *p* = 0.017). Also, plasma SAM levels correlated with BMI (r = 0.51, *p* = 0.007) and whole-body fat mass (r = 0.56, *p* = 0.005).

## Discussion

Here, we identified genome-wide expression patterns in liver biopsies collected from 26 severely obese individuals. The patients represented various degrees of NAFLD and were categorized based on NAS, ranging from no/mild NAFLD (NAS 0–1), moderate NAFLD (NAS 2–3) to more advanced NAFLD (NAS 4–6). Hepatic transcriptome signatures in patients with moderate and more advanced NAFLD were characterized by increased gene expression of markers of stimulated immune system activity and pro-fibrotic signaling, clearly separating individuals with no/mild NAFLD from individuals with moderate and more advanced NAFLD. Among significantly regulated genes fulfilling criteria as potentially liver-selective and with a secreted gene product, we selected four candidate genes with biomarker potential (*LPA*, *IGFBP-1*, *SERPINF2*, *MAT1A*) and measured plasma concentrations of the corresponding gene products (LPA, IGFBP-1, α2AP and SAM). Even though circulating concentrations of these gene products were unable to discriminate among the different NAFLD categories in the present cohort, this novel approach for defining hepatic secretome gene signatures in NAFLD patients may be instrumental for identification of future biomarker candidates to aid in the diagnosis and treatment of NAFLD in an unbiased and efficient manner.

All four selected hepatic gene biomarker candidates were differentially expressed in individuals with moderate to more advanced NAFLD compared to no/mild NAFLD. This finding was subsequently validated in three large, publicly available datasets of NAFLD patients [13–15]. Notably, candidate genes displayed high levels of expression in the liver compared to other tissues. Whereas robust downregulation of these four candidate genes was detected in the present study and confirmed in independent NAFLD studies, the gene products in plasma were not regulated in the corresponding patients. Correlation plots confirmed lack of correlation between candidate gene expression levels and concentrations of the corresponding gene products in plasma. The discrepancy between transcript and protein levels in plasma is not uncommon and has previously been described [29–31]. Accordingly, various post-transcriptional mechanisms are involved in the regulation of secretion and plasma stability of proteins which may explain the discrepancy between transcriptomics data and circulating levels of proteins in the present study [29, 30].

The biological function of the four gene products has previously been established. Accordingly, LPA is a cholesteryl ester-rich lipoprotein and is a well-known risk factor of cardiovascular disease [32]. IGFBP-1 is a binding protein of insulin-like growth factors modulating their anabolic activity [33]. α2AP (*SERPINF2*) is the principal inhibitor of plasmin, which is responsible for fibrinolysis [34] and has been investigated as a target for treatment of thrombotic diseases [35]. Conversely, increased activation of plasmin secondary to lowered plasma α2AP levels has been proposed to play a role in progression of liver fibrosis in non-NASH settings [36]. Finally, SAM (*MAT1A*) is known as the main biological methyl donor and is required for a number of biochemical reactions including methylation of phospholipids, a crucial step in lipid metabolism [37]. In support of the functional relevance of SAM/MAT1A in metabolic disease, MAT1A-knockout mice show NASH and dyslipidemia [38]. All four proteins are produced in the liver as products of their respective liver-expressed genes [34, 37, 39, 40]. Plasma concentrations of LPA and IGFBP-1 have previously been reported to correlate with hepatic mRNA levels [39, 40], while no association between gene expression levels of *SERPINF2* and α2AP protein levels was found in hepatitis C virus-infected patients [36]. To the best of our knowledge, comparative studies on plasma concentrations of SAM and hepatic expression levels of *MAT1A* have not been reported. However, it is well-established that decreased *MAT1A* expression leads to impaired synthesis of SAM [37]. The previously reported correlation between hepatic expression levels and plasma concentrations of LPA [39] and IGFBP-1 [40] could not be confirmed. The discrepancy may be due to differences in study populations and/or the limited power of the present study.

Previous clinical studies have linked NAFLD to each of the four candidate genes/gene products. For example, decreased plasma concentrations of LPA and reduced hepatic expression of *LPA* have been associated with NAFLD progression and advanced hepatic fibrosis in NAFLD patients [39]. In addition, decreased circulating levels of LPA have been correlated with declining liver function in other chronic liver diseases [41, 42]. Likewise, decreased circulating levels of α2AP have been associated with advanced liver fibrosis in two studies characterizing the plasma proteome in NAFLD patients and controls [9, 43], and decreased plasma concentrations of SAM and hepatic expression levels of *MAT1A* have been associated with NAFLD and/or liver fibrosis [12, 44, 45]. In agreement with previous findings, we confirmed that decreased hepatic expression levels of *LPA* and *MAT1A* are associated with the severity of NAFLD [12, 39]. Previous studies have pointed to an inverse relationship between plasma concentrations of IGFBP-1 and the liver fat content (based on proton magnetic resonance spectroscopy) [46] whereas higher plasma concentrations have been reported in individuals with more advanced liver fibrosis (stage 3–4) compared to no or less advanced fibrosis (stage 0–2) [47]. Furthermore, increased hepatic IGFBP-1 expression (based on immunohistochemistry) was found in individuals with NAFL compared to healthy controls [48]. To our knowledge, neither *IGFBP-1* nor *SERPINF2* gene expression has previously been investigated in individuals with various degrees of NAFLD, and the few existing studies concerning these candidate genes/gene products are further limited by missing comparison of expression levels and plasma protein concentrations. Insulin resistance is a hallmark in the pathogenesis of NAFLD pathogenesis [49]. Notably, both LPA and IGFBP-1 have been suggested to be inversely associated with insulin resistance [39, 40]. This finding was not confirmed in the present study, as assessed by HOMA, although a correlation between IGFBP-1 and C-peptide was observed. Both IGFBP-1 and SAM have been explored as potential therapeutic targets for NAFLD in rodent and *in vitro* models, improving histological hallmarks of NAFLD [38, 48]. Moreover, administration of SAM has been investigated in patients with alcoholic liver disease but without any effects on biochemical or histopathological parameters [50]. Even though SAM remains to be tested in individuals with NAFLD, there is an increasing interest in both *MAT1A* and SAM as treatment targets for NASH [37].

Liver biopsy transcriptomics in patients with NAFLD has provided important insight into molecular determinants of NAFLD, which may potentially guide development of novel biomarkers [12–15]. Two recently published studies have carefully characterized liver transcriptional changes across the entire NAFLD spectrum by RNA sequencing to identify novel biomarkers and treatment targets from selected DEGs [14, 15]. However, as exemplified in the current study, hepatic gene expression changes are not necessarily reflected by corresponding systemic regulations in gene product levels which makes it challenging to interpret hepatic gene expression changes in the context of developing biomarkers of NAFLD [31]. Accordingly, Govaere et al. [14] applied proteomics analysis of NAFLD serum samples to determine if a gene set consisting of 25 DEGs could be detected in the circulation. Only 14 genes encoded circulating proteins, and out of these, only five proteins correlated with one or more histological features of NAFLD. Together with our data, this underscores the need for bioinformatics approaches to better identify hepatic gene signatures associated with the liver secretome to facilitate identification of candidate genes as potential biomarkers of NAFLD/NASH.

Limitations of the present study should be considered. Firstly, the selected candidate gene products were measured by ELISA, which may only detect one peptide isoform. Additional quantitative protein assays such as mass spectrometry should be employed to distinguish between potential isoforms of the plasma proteins analyzed in the present study. Secondly, the small sample size of 26 liver biopsies may have limited the statistical power to detect DEGs related to NAFLD progression, i.e. the number of DEGs is probably underestimated. Thirdly, the present cohort does not represent the whole spectrum of NAFLD. Hence, the relatively few cases of histologically verified NASH and fibrosis may have limited findings of candidate genes relevant for NAFLD. Fourthly, we chose to divide participants into three groups based on relatively narrow NAS intervals because of few cases with definite histopathological signs of NASH and/or severe fibrosis. Since NAS reflects the composite, unweighted sum of steatosis, lobular inflammation and ballooning degeneration, it could be argued that NASH vs. NAFL or no fibrosis vs. liver fibrosis would represent more clinically relevant categorizations of participants. Nevertheless, the biomarker candidate genes identified in the present study were confirmed in three large, independent cohorts of NAFLD patients [13–15]. It should be considered that the present study is a part of a prospective, single-site study aiming to investigate the effects of bariatric surgery on NAFLD. In this regard, the patient cohort was carefully characterized by clinical, anthropometrical and biochemical examinations including a DXA scan for evaluation of body composition, and the biopsy procedures were performed by the same two investigators. We speculate that the present approach applied to paired and prospective liver biopsy data from patients either developing NAFLD or experiencing remission of NAFLD (i.e., within-subject changes) may, potentially, provide novel circulating biomarker candidates of NAFLD.

In conclusion, we have established a bioinformatics pipeline allowing for predicting and characterizing potential circulating biomarkers based on liver secretome gene signatures in severely obese patients with histologically verified NAFLD. Using this approach in larger NAFLD patient cohorts may further identify liver secretome gene signatures encoding potential, circulating biomarkers for NAFLD.

## Supporting information

S1 Fig Progression of NAFLD is accompanied by increased proportional area of steatosis and fibrosis. Histological quantification of steatosis and fibrosis. Proportional (%) area of lipids (HE staining) and fibrosis (PSR staining). * *p* <0.05, ** *p* <0.01 (Dunn’s post hoc test compared to NAS 0–1). Abbreviations: HE, hematoxylin-eosin; NAFLD; non-alcoholic fatty liver disease; NAS, NAFLD activity score; PSR, picro-Sirius Red.
(PDF)

S2 Fig Immunohistochemical markers of inflammation, macrophage infiltration and fibrogenesis. Left panels, proportional (%) area of immunohistological marker. Middle panels, expression levels of the corresponding gene. Right panels, correlation plot of histological marker vs. corresponding gene. **(A)** Galectin-3. **(B)** CD11b. **(C)** CD45. **(D)** Alpha-smooth muscle actin (α-SMA). **(E)** Collagen-1a1 (Col1A1). Abbreviations: NAS, non-alcoholic fatty liver disease activity score.
(PDF)

S3 Fig Representative photomicrographs of galectin-3 (Gal3), CD11b, CD45, alpha-smooth muscle actin (α-SMA) and collagen-1a1 (Cola1a1). (PDF)

S4 Fig Validation of human NAFLD candidate genes in three large, publicly available datasets. GEO accession numbers: **(A)** GSE162694 (Pantano et al., Sci Rep 11:1804, 2021). **(B)** GSE135251 (Govaere et al., Sci. Transl. Med. 12: eaba4448, 2020). **(C)** GSE130970 (Hoang et al., Sci Rep. 9:12541, 2019). * *p* <0.05, ** *p* <0.01, *** *p* <0.001 vs. NAS 0–1. Abbreviations: NAS, non-alcoholic fatty liver disease activity score.
(PDF)

S5 Fig Expression levels of candidate genes and albumin across tissues. Gene expression data were evaluated from the human protein atlas (Humphery-Smith et al., Proteomics 4:2519–2521, 2004). Abbreviations: ALB, albumin; IGFBP-1, insulin-like growth factor-binding protein 1; LPA, lipoprotein A; MAT1A, methionine adenosyltransferase 1A; SERPINF2, serpin family F member 2; TPM, transcript per million.
(PDF)

S6 Fig Correlation plots of hepatic candidate gene expression levels and plasma concentrations of the corresponding gene product. **(A)** Insulin-like growth factor-binding protein 1 (IGFBP-1). **(B)** Methionine adenosyltransferase 1A (MAT1A) and enzyme catalytic product (S-adenosylmethionine (SAM)). **(C)** Lipoprotein A (LPA). **(D)** Serpin family F member 2 (SERPINF2) and gene product alpha-2 antiplasmin (α2AP).
(PDF)

S1 Table Additional anthropometrical and biochemical characteristics of study participants divided into three groups with increasing NAFLD activity score. Data are presented as median with interquartile range in parentheses unless otherwise stated. *P* values are Kruskal-Wallis tests. * *p* <0.05; ** *p* <0.01 (Dunn’s post-hoc test, compared to NAS 0–1) Abbreviations: NAFLD, non-alcoholic fatty liver disease; NAS, NAFLD activity score; AUDIT-C, alcohol use disorders identification test.
(PDF)

## Abbreviations

NAFLD: non-alcoholic fatty liver disease
LPA: lipoprotein A
IGFBP-1: insulin-like growth factor-binding protein 1
SERPINF2: serpin family F member 2
MAT1A: methionine adenosyltransferase 1A
NASH: non-alcoholic steatohepatitis
NAFL: non-alcoholic fatty liver; NAS, NAFLD activity score
BMI: body mass index
DXA: dual-energy X-ray absorptiometry
DEGs: differentially expressed genes
α2AP: alpha-2 antiplasmin
SAM: S-adenosylmethionine
HE: hematoxylin-eosin
PSR: picro-Sirius Red
α-SMA: alpha-smooth muscle action
COL1A1: anti-type I collagen
HOMA: homeostasis model assessment
PCA: principal component analysis
