# Supplementary material for: A liver secretome gene signature-based approach for determining circulating biomarkers of NAFLD severity
Source: PLoS One. 2022 Oct 19;17(10):e0275901. doi: 10.1371/journal.pone.0275901 (PMC9581378; doi:10.1371/journal.pone.0275901)
Supplement: S1 Fig — Histological quantification of steatosis and fibrosis. Proportional (%) area of lipids (HE staining) and fibrosis (PSR staining). * p <0.05, ** p <0.01 (Dunn’s post hoc test compared to NAS 0–1). Abbreviations: HE, hematoxylin-eosin; NAFLD; non-alcoholic fatty liver disease; NAS, NAFLD activity score; PSR, picro-Sirius Red. (PDF) [file pone.0275901.s001.pdf]

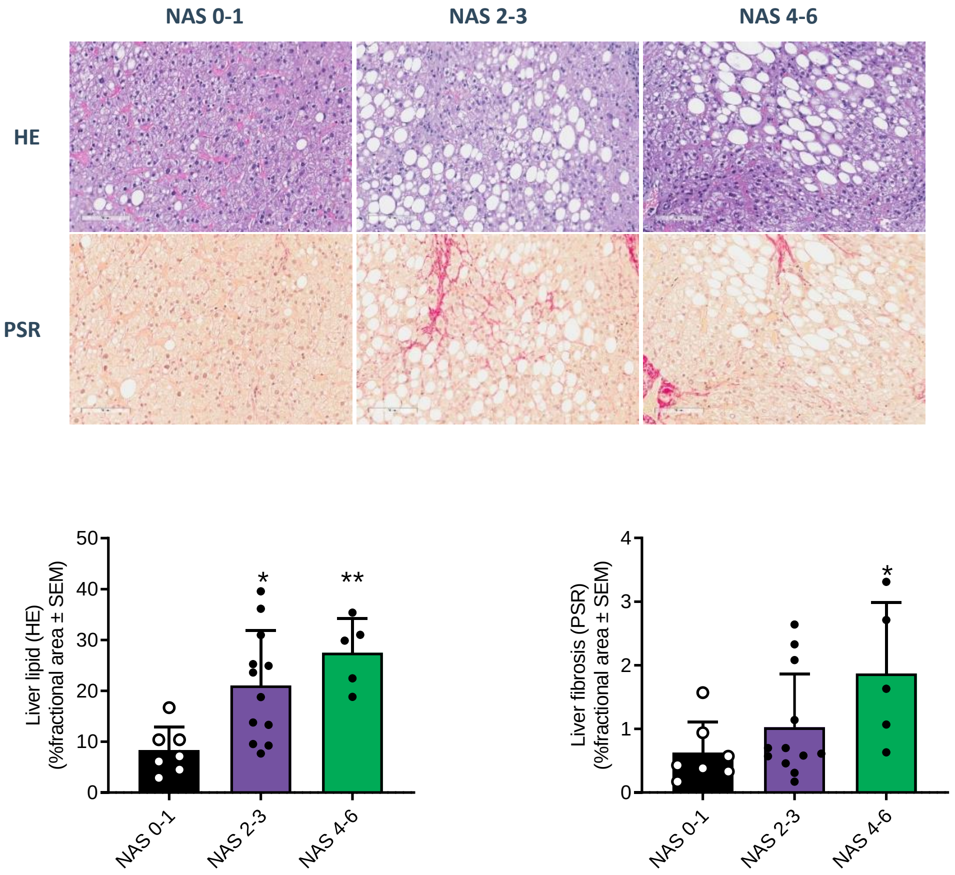

S1 Fig. Progression of NAFLD is accompanied by increased proportional area of steatosis and fibrosis. Histological quantification of steatosis and fibrosis. Proportional (%) area of lipids (HE staining) and fibrosis (PSR staining). * *p* <0.05, ** *p* <0.01 01 (Dunn's post hoc test compared to NAS 0-1). Abbreviations: HE, hematoxylin-eosin; NAFLD; non-alcoholic fatty liver disease; NAS, NAFLD activity score; PSR, picro-Sirius Red.
