# Supplementary material for: A liver secretome gene signature-based approach for determining circulating biomarkers of NAFLD severity
Source: PLoS One. 2022 Oct 19;17(10):e0275901. doi: 10.1371/journal.pone.0275901 (PMC9581378; doi:10.1371/journal.pone.0275901)
Supplement: S2 Fig — Left panels, proportional (%) area of immunohistological marker. Middle panels, expression levels of the corresponding gene. Right panels, correlation plot of histological marker vs. corresponding gene. (A) Galectin-3. (B) CD11b. (C) CD45. (D) Alpha-smooth muscle actin (α-SMA). (E) Collagen-1a1 (Col1A1). Abbreviations: NAS, non-alcoholic fatty liver disease activity score. (PDF) [file pone.0275901.s002.pdf]

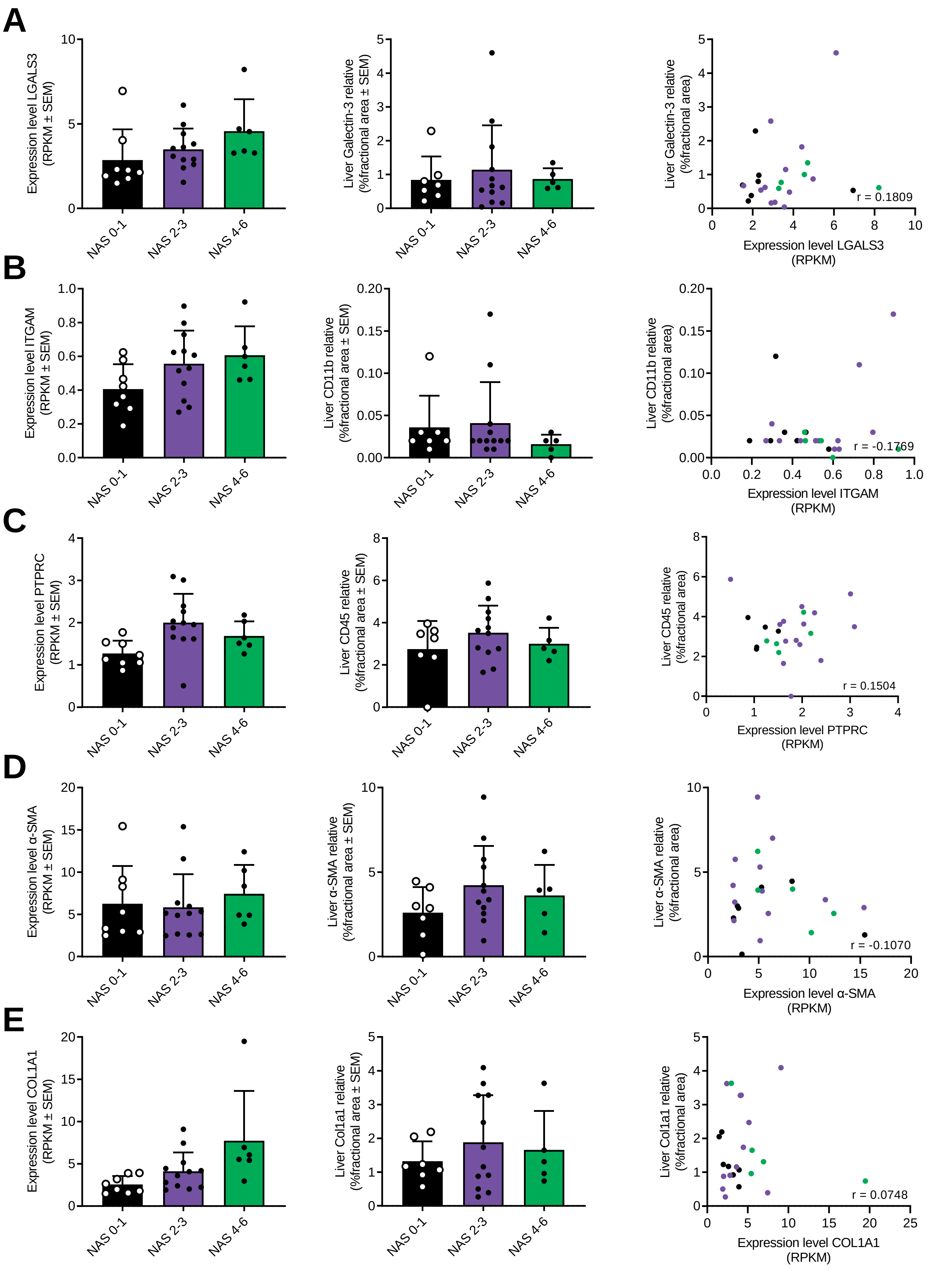

S2 Fig. Immunohistochemical markers of inflammation, macrophage infiltration and fibrogenesis. Left panels, proportional (%) area of immunohistological marker. Middle panels, expression levels of the corresponding gene. Right panels, correlation plot of histological marker vs. corresponding gene. **(A)** Galectin-3. **(B)** CD11b. **(C)** CD45. **(D)** Alpha-smooth muscle actin (α-SMA). **(E)** Collagen-1a1 (Col1A1). Abbreviations: NAS, non-alcoholic fatty liver disease activity score.
