# Supplementary material for: A liver secretome gene signature-based approach for determining circulating biomarkers of NAFLD severity
Source: PLoS One. 2022 Oct 19;17(10):e0275901. doi: 10.1371/journal.pone.0275901 (PMC9581378; doi:10.1371/journal.pone.0275901)
Supplement: S3 Fig — (PDF) [file pone.0275901.s003.pdf]

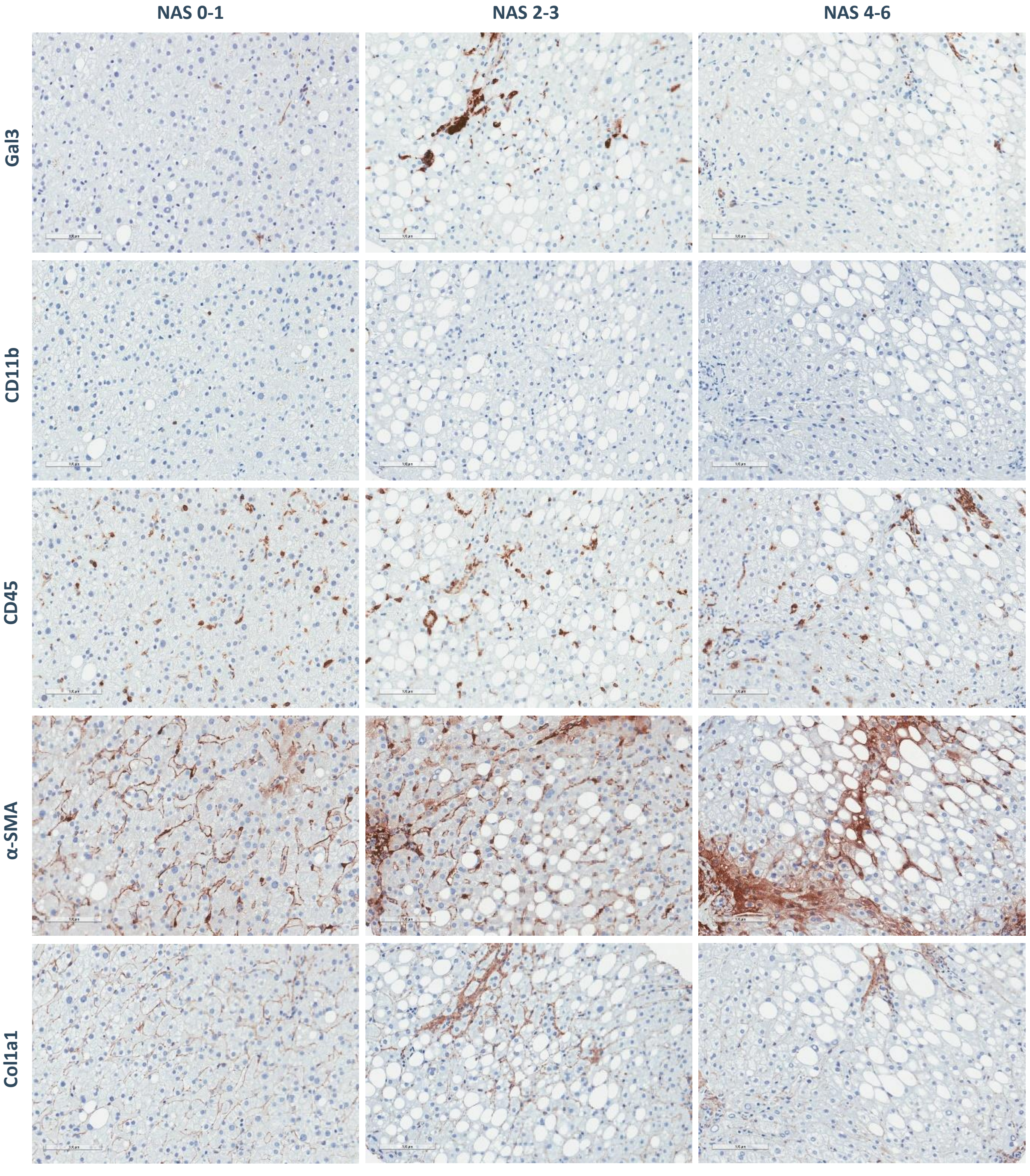

S3 Fig. Representative photomicrographs of galectin-3 (Gal3), CD11b, CD45, alpha-smooth muscle actin (α-SMA) and collagen-1a1 (Cola1a1).
