# Supplementary material for: A liver secretome gene signature-based approach for determining circulating biomarkers of NAFLD severity
Source: PLoS One. 2022 Oct 19;17(10):e0275901. doi: 10.1371/journal.pone.0275901 (PMC9581378; doi:10.1371/journal.pone.0275901)
Supplement: S4 Fig — GEO accession numbers: (A) GSE162694 (Pantano et al., Sci Rep 11:1804, 2021). (B) GSE135251 (Govaere et al., Sci. Transl. Med. 12: eaba4448, 2020). (C) GSE130970 (Hoang et al., Sci Rep. 9:12541, 2019). * p <0.05, ** p <0.01, *** p <0.001 vs. NAS 0–1. Abbreviations: NAS, non-alcoholic fatty liver disease activity score. (PDF) [file pone.0275901.s004.pdf]

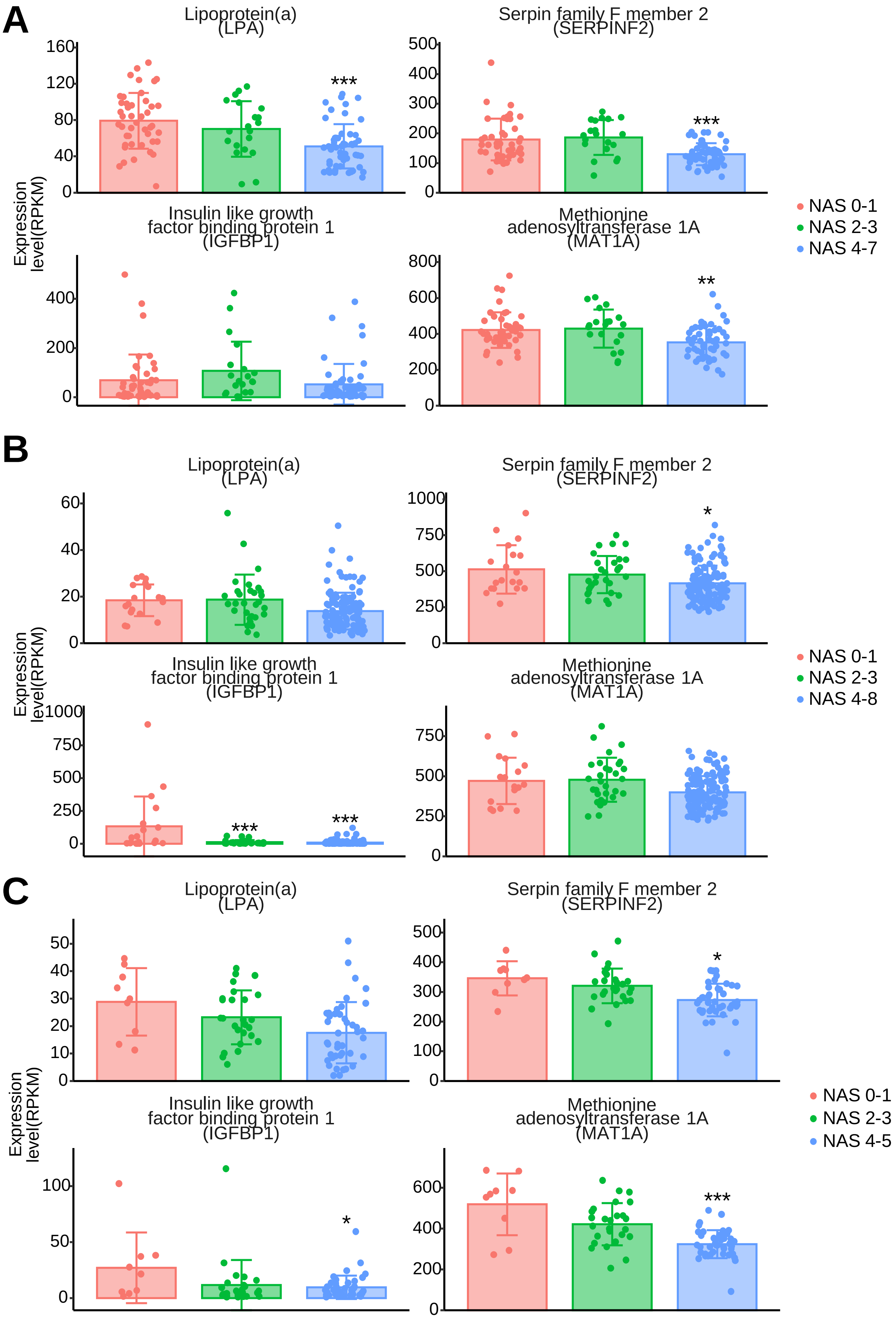

S4 Fig. Validation of human NAFLD candidate genes in three large, publicly available datasets. GEO accession numbers: (A) GSE162694 (Pantano et al., Sci Rep 11:1804, 2021). **(B)** GSE135251 (Govaere et al., Sci. Transl. Med. 12: eaba4448, 2020). **(C)** GSE130970 (Hoang et al., Sci Rep. 9:12541, 2019). * p <0.05, ** p <0.01, *** p <0.001 vs. NAS 0–1. Abbreviations: NAS, non-alcoholic fatty liver disease activity score.
