# Supplementary material for: A liver secretome gene signature-based approach for determining circulating biomarkers of NAFLD severity
Source: PLoS One. 2022 Oct 19;17(10):e0275901. doi: 10.1371/journal.pone.0275901 (PMC9581378; doi:10.1371/journal.pone.0275901)
Supplement: S5 Fig — Gene expression data were evaluated from the human protein atlas (Humphery-Smith et al., Proteomics 4:2519–2521, 2004). Abbreviations: ALB, albumin; IGFBP-1, insulin-like growth factor-binding protein 1; LPA, lipoprotein A; MAT1A, methionine adenosyltransferase 1A; SERPINF2, serpin family F member 2; TPM, transcript per million. (PDF) [file pone.0275901.s005.pdf]

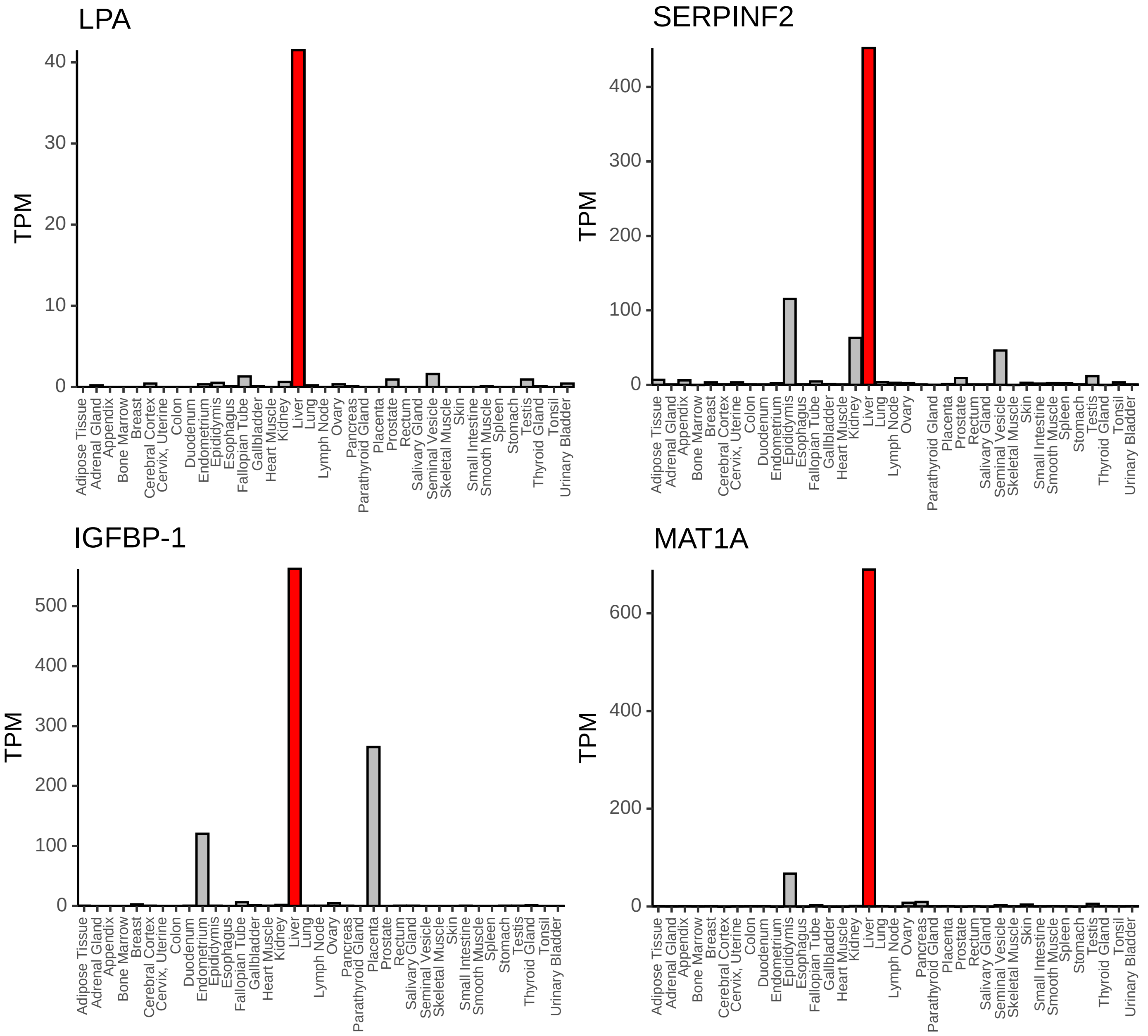

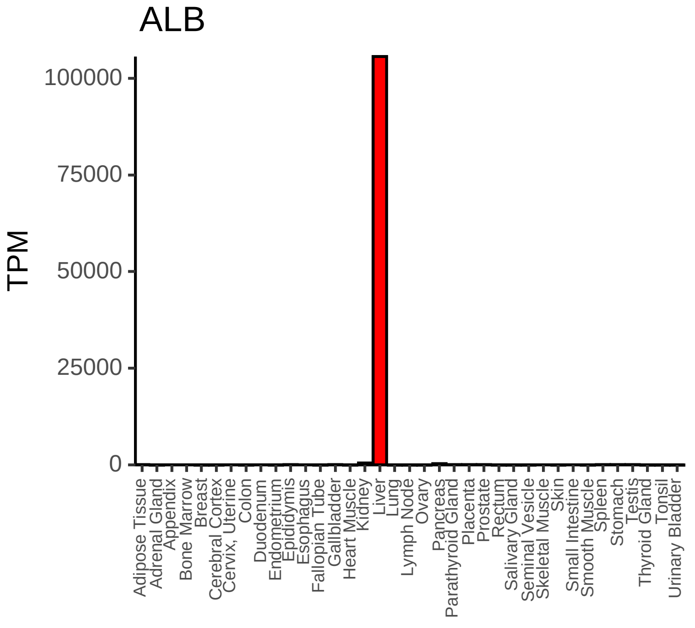

S5 Fig. Expression levels of candidate genes and albumin across tissues. Gene expression data were evaluated from the human protein atlas (Humphery-Smith et al., Proteomics 4:2519–2521, 2004). Abbreviations: ALB, albumin; IGFBP-1, insulin-like growth factor-binding protein 1; LPA, lipoprotein A; MAT1A, methionine adenosyltransferase 1A; SERPINF2, serpin family F member 2; TPM, transcript per million.
