# Supplementary material for: A liver secretome gene signature-based approach for determining circulating biomarkers of NAFLD severity
Source: PLoS One. 2022 Oct 19;17(10):e0275901. doi: 10.1371/journal.pone.0275901 (PMC9581378; doi:10.1371/journal.pone.0275901)
Supplement: S6 Fig — (A) Insulin-like growth factor-binding protein 1 (IGFBP-1). (B) Methionine adenosyltransferase 1A (MAT1A) and enzyme catalytic product (S-adenosylmethionine (SAM)). (C) Lipoprotein A (LPA). (D) Serpin family F member 2 (SERPINF2) and gene product alpha-2 antiplasmin (α2AP). (PDF) [file pone.0275901.s006.pdf]

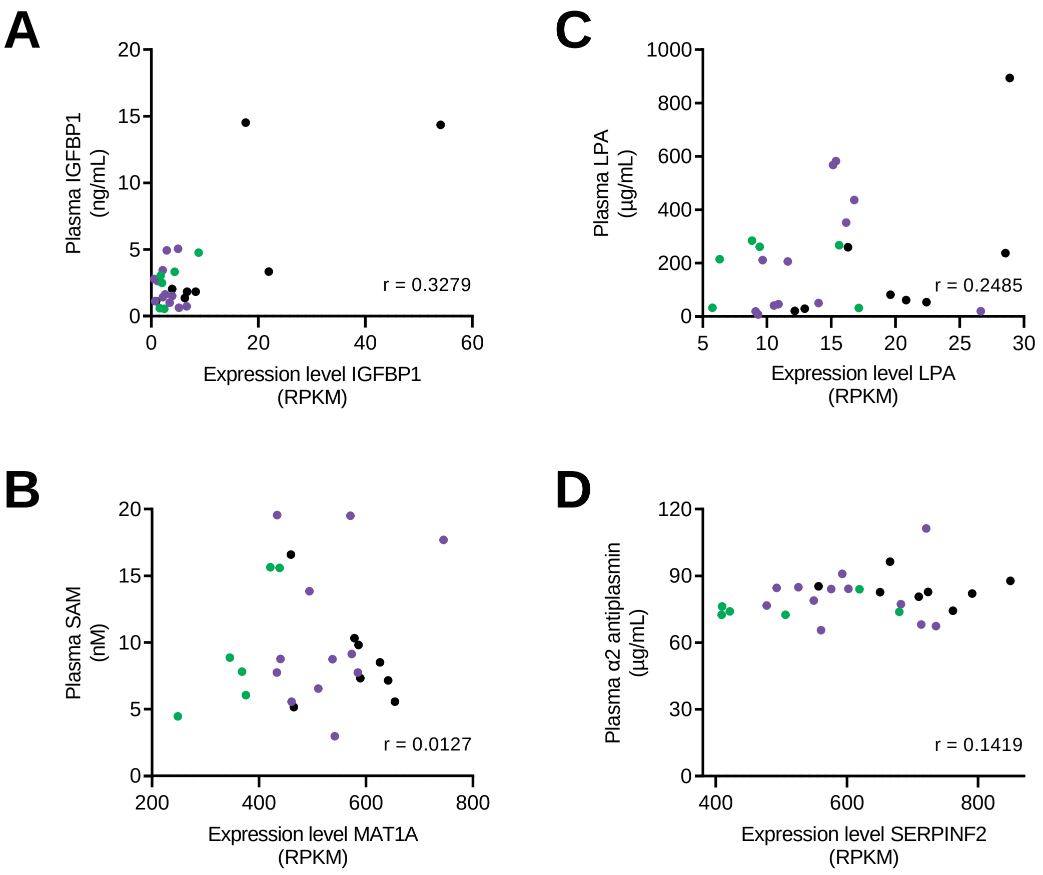

S6 Fig. Correlation plots of hepatic candidate gene expression levels and plasma concentrations of the corresponding gene product. (A) Insulin-like growth factor-binding protein 1 (IGFBP-1). **(B)** Methionine adenosyltransferase 1A (MAT1A) and enzyme catalytic product (S-adenosylmethionine (SAM)). **(C)** Lipoprotein A (LPA). **(D)** Serpin family F member 2 (SERPINF2) and gene product alpha-2 antiplasmin (α2AP).
