# Supplementary material for: A liver secretome gene signature-based approach for determining circulating biomarkers of NAFLD severity
Source: PLoS One. 2022 Oct 19;17(10):e0275901. doi: 10.1371/journal.pone.0275901 (PMC9581378; doi:10.1371/journal.pone.0275901)
Supplement: S1 Table — Data are presented as median with interquartile range in parentheses unless otherwise stated. P values are Kruskal-Wallis tests. * p <0.05; ** p <0.01 (Dunn’s post-hoc test, compared to NAS 0–1) Abbreviations: NAFLD, non-alcoholic fatty liver disease; NAS, NAFLD activity score; AUDIT-C, alcohol use disorders identification test. (PDF) [file pone.0275901.s007.pdf]

Table S1. Additional anthropometrical and biochemical characteristics of study participants divided into three groups with increasing NAFLD Activity Score

	**NAS 0-1 (*n* = 8)**	**NAS 2-3 (*n* = 12)**	**NAS 4-6 (*n* = 6)**	***p* value**
Fat free mass, bioimpedance analysis (kg)	77.8 (54.4;82.7)	67.2 (61.8;80.8)	83.7 (73.2;91.2)	0.375
Muscle mass, bioimpedance (kg)	38.8 (26.5;42.4)	34.4 (30.8;41.0)	42.9 (38.0;47.3)	0.257
Fat mass, bioimpedance (kg)	61.0 (52.9;66.9)	78.2 (72.1;87.4)*	62.2 (59.0;74.8)	0.012
Body fat, bioimpedance analysis (%)	45.5 (43.9;51.0)	54.4 (50.6;56.0)**	46.0 (40.5;49.0)	0.005
Platelet count (E^9/L)	300 (254;317)	324 (227;376)	270 (243;306)	0.374
Albumin (g/L)	39 (37;40)	41 (41;43)*	39 (39;42)	0.023
Alkaline phosphatase (U/L)	93 (79;112)	77 (63;84)	81 (71;90)	0.092
Lactate dehydrogenase (U/L)	176 (156;194)	212 (185;226)*	235 (195;263)**	0.005
Bilirubin (µmol/L)	9 (7;13)	9 (8;11)	10 (5;12)	0.922
Ionized calcium (mmol/L)	1.2 (1.2;1.3)	1.2 (1.2;1.3)	1.2 (1.2;1.3)	0.555
Phosphate (mmol/L)	1.1 (1.0;1.2)	1.0 (0.9;1.1)	1.0 (0.8;1.9)	0.763
Carbamide (mmol/L)	5.2 (4.0;6.0)	3.8 (3.3;5.3)	3.9 (3.7;4.9)	0.236
Vitamin B_{12} (pmol/L)	384 (338;528)	394 (281;619)	422 (340;505)	0.854
25-hydroxy vitamin D (nmol/L)	67 (46;119)	69 (47;82)	63 (45;80)	0.836
Parathyroid hormone (pmol/L)	5.8 (5.3;7.0)	8.8 (6.5;12.2)	6.2 (5.4;7.9)	0.076
Thyroid-stimulating hormone (IU/L)	1.6 (1.3;2.2)	1.6 (1.1;2.6)	2.8 (1.4;3.9)	0.344
Average alcohol units per week	2 (0;8)	1 (0;1)	2 (0;6)	0.547
AUDIT-C (19)	4 (1;5)	3 (1;4)	3 (3;6)	0.516

Data are presented as median with interquartile range in parentheses unless otherwise stated. *P* values are Kruskal-Wallis tests. * $p < 0.05$; ** $p < 0.01$ (Dunn's post-hoc test, compared to NAS 0-1) Abbreviations: NAFLD, non-alcoholic fatty liver disease; NAS, NAFLD activity score; AUDIT-C, alcohol use disorders identification test.
